# Health-Related Quality of Life in Patients with Spinocerebellar Ataxia: a Validation Study of the EQ-5D-3L

**DOI:** 10.1007/s12311-023-01597-3

**Published:** 2023-09-15

**Authors:** Maresa Buchholz, Niklas Weber, Anika Rädke, Jennifer Faber, Tanja Schmitz-Hübsch, Heike Jacobi, Feng Xie, Thomas Klockgether, Bernhard Michalowsky, The EUROSCA study group, Sophie Tezenas du Montcel, Peter Bauer, Paola Giunti, Arron Cook, Robyn Labrum, Michael H. Parkinson, Alexandra Durr, Alexis Brice, Perrine Charles, Cecilia Marelli, Caterina Mariotti, Lorenzo Nanetti, Marta Panzeri, Maria Rakowicz, Anna Sulek, Anna Sobanska, Ludger Schöls, Holger Hengel, Laszlo Baliko, Bela Melegh, Alessandro Filla, Antonella Antenora, Jon Infante, José Berciano, Bart P. van de Warrenburg, Dagmar Timmann, Sandra Szymanski, Sylvia Boesch, Jun-Suk Kang, Massimo Pandolfo, Jörg B. Schulz, Sonia Molho, Alhassane Diallo, The ESMI study group, Jeanette Hübener-Schmid, Magda Santana, Marcus Grobe-Einsler, Berkan Koyak, Mafalda Raposo, Manuela Lima, Hector Garcia-Moreno, Paola Giunti, Luís Pereira de Almeida, Bart van de Warrenburg, Judith van Gaalen, Dagmar Timmann, Andreas Thieme, Kathrin Reetz, Imis Dogan, Carlo Wilke, Ludger Schöls, Olaf Riess, Matthis Synofzik, Jeroen de Vries, Jon Infante, Oz Gulin, James Joers, Chiadikaobi Onyike, Michal Povazan, Eva-Maria Ratai, Jeremy Schmahmann

**Affiliations:** 1 Translational Health Care Research, German Center for Neurodegenerative Diseases (DZNE), Site Rostock/Greifswald, Greifswald, Germany; 2 German Center for Neurodegenerative Diseases (DZNE), Bonn, Germany; 3 Department of Neurology, University Hospital Bonn, Bonn, Germany; 4 Experimental and Clinical Research Center, a cooperation of Max-Delbrueck Center of Molecular Medicine and Charité – Universitätsmedizin Berlin, Berlin, Germany; 5 Department of Neurology, University Hospital Heidelberg, Heidelberg, Germany; 6 Department of Health Research Methods, Evidence and Impact, McMaster University, Hamilton, Ontario, Canada

**Keywords:** Validation, EQ-5D-3L, Health-related quality of life, Spinocerebellar ataxia

## Abstract

Although health-related quality of life (HRQoL) has developed into a crucial outcome parameter in clinical research, evidence of the EQ-5D-3L validation performance is lacking in patients with spinocerebellar ataxia (SCA) types 1, 2, 3, and 6. The objective of this study is to assess the acceptability, validity, reliability, and responsiveness of the EQ-5D-3L. For *n* = 842 predominantly European SCA patients of two longitudinal cohort studies, the EQ-5D-3L, PHQ-9 (Patient Health Questionnaire), and ataxia-specific clinical assessments (SARA: Scale for Assessment and Rating of Ataxia; ADL: activities of daily living as part of Friedreich’s Ataxia Rating Scale; INAS: Inventory of Non-Ataxia Signs) were assessed at baseline and multiple annual follow-ups. The EQ-5D-3L was evaluated regarding acceptability, distribution properties, convergent and known-groups validity, test-retest reliability, and effect size measures to analyze health changes. The non-item response was low (EQ-5D-3L index: 0.8%; EQ-VAS: 3.4%). Ceiling effects occurred in 9.9% (EQ-5D-3L) and 3.0% (EQ-VAS) with a mean EQ-5D-3L index of 0.65 ± 0.21. In total, convergent validity showed moderate to strong Spearman’s rho (*r*_s_ > 0.3) coefficients comparing EQ-5D-3L and EQ-VAS with PHQ-9, SARA, ADL, and INAS. EQ-5D-3L could discriminate between groups of age, SARA, ADL, and INAS. Intra-class correlation coefficients (EQ-5D-3L_ICC_: 0.95/EQ-VAS_ICC_: 0.88) and Kappa statistics (range 0.44 to 0.93 for EQ-5D-3L items) indicated tolerable reliability. EQ-5D-3L shows small (effect size < 0.3) to moderate (effect size 0.3–0.59) health changes regarding ataxia severity. The analysis confirms an acceptable, reliable, valid, and responsive recommended EQ-5D-3L in SCA patients, measuring the HRQoL adequately, besides well-established clinical instruments.

**Supplementary Information:**

The online version contains supplementary material available at 10.1007/s12311-023-01597-3.

## Introduction

Spinocerebellar ataxias (SCAs) comprise a heterogeneous group of autosomal dominantly inherited diseases caused by degeneration of the cerebellum and its connections [1]. SCAs have an estimated global prevalence of 3–4 per 100,000. SCA 1, SCA 2, SCA 3, and SCA 6 account for more than half of the affected families [2, 3].

SCAs commonly manifest in the third and fourth decades of life [4] and are characterized by prominent ataxia often accompanied by additional neurological symptoms. Cognitive impairment and depression may also occur [4]. Patients with SCA have a shorter life expectancy and suffer substantial limitations in their daily activities. The disease-specific functional limitations and the emotional burdens harm the patient’s health-related quality of life (HRQoL) [5].

Measuring HRQoL has become a crucial outcome in patients with chronic conditions to assess their subjective health perspective in clinical trials or to optimize treatment decision-making [6, 7]. Epstein et al. [8] referred in their work about self-rated health in Friedreich ataxia (FA) that HRQoL measures were potentially useful as clinical markers of the ataxia disease status. Only a few previous studies evaluated the impact of SCA on a patient’s HRQoL, revealing a significant decline over time and associations with ataxia severity, functional impairment, pain, depressive symptoms, and fatigue [9–11].

However, evidence about the validity of appropriate HRQoL measures in SCAs is missing. The National Institute for Health and Care Excellence (NICE) recommends using the widely used and well-established generic preference-based EQ-5D in rare diseases [12]. Only one study focused on the psychometric properties of the EQ-5D-3L in patients with Friedreich ataxia [13]. Based on a sample of *n* = 56 patients, the authors rated the EQ-5D-3L as a measure with poor discriminative ability. However, the results of this cross-sectional study with a small sample size remain inconclusive and should be interpreted with caution. Further validation studies are needed to generate more robust results concerning the psychometric performance of HRQoL instruments in SCA.

This analysis aimed to assess the psychometric properties of the EQ-5D-3L in terms of acceptability, validity, reliability, and responsiveness. To this end, EQ-5D-3L from large samples of patients with SCA 1, 2, 3, and 6 were analyzed and followed up annually for several years in different observational multi-center studies. This large data set of a rare disorder allows us to validate the EQ-5D-3L longitudinally on an appropriate sample size for the first time.

## Patients and Methods

### Study Design, Recruitment, and Sample

We analyzed data from two prospective, longitudinal observational ataxia cohort studies carried out at several European and US study centers: [1] European Spinocerebellar Ataxia Type 3/Machado-Joseph Disease Initiative (ESMI) and [2] European Spinocerebellar Ataxia Registry (EUROSCA). ESMI is an ongoing, longitudinal cohort study of SCA 3 mutation carriers and controls. The cohort started in 2016 and is still ongoing in its 5th year of recruitment. Both recruitment and follow-ups take place in 11 European (majority of study participants) and three US study sites [14]. EUROSCA is a European longitudinal study with people with manifest SCA 1, 2, 3, and 6 carried out in 17 European sites [15, 16]. The study was initiated in 2005, and participants were consecutively recruited and followed up until 2016 [15]. ESMI and EUROSCA were approved by the ethics committees of the participating centers and have been performed in accordance with the ethical standards laid down in the 1964 Declaration of Helsinki and its later amendments. Written informed consent was obtained from all study participants. EUROSCA was registered with a ClinicalTrials.gov number (NCT02440763).

In both studies, clinical visits were scheduled annually within the first 3 years after baseline (*T*_0_ = baseline, *T*_1_ = follow-up 1, *T*_2_ = follow-up 2, *T*_3_ = follow-up 3). We checked for time between observations (*T*_0_−*T*_1_, *T*_1_−*T*_2_, and *T*_2_−*T*_3_), providing mean, standard deviation, and interquartile range in supplementary Table 1. Afterwards, participants entered an extension phase in which study visits were scheduled in combination with routine visits resulting in irregular intervals. Therefore, only data from baseline to *T*_3_ were included in this analysis. The flow chart in Supplementary Figure 1 illustrates patient selection from both studies.

SCA 1, 2, and 3 present a similar clinical picture with an age onset between 30 and 40 years and frequently more non-ataxia symptoms [16, 17]. Patients suffering from SCA 6 are older (> 60 years old), show a slower disease progression, and have less additional non-ataxia signs, revealing SCA 6 as a purely cerebellar disorder [16, 17]. Based on these aspects, patients of both studies were separated into two groups: patients with SCA 1, 2, or 3 vs. patients with SCA 6.

### Measures

In both studies, patients completed a set of socio-demographics (age, sex), age of onset of gait difficulties, clinical, and health-related questionnaires at baseline and every follow-up. The EQ-5D-3L and the Patient Health Questionnaire 9 (PHQ-9) were administered as health-related questionnaires. Rater-based clinical ataxia-specific measures were the Scale for Assessment and Rating of Ataxia (SARA) and the activities of daily living (ADL) as part of Friedreich’s Ataxia Rating Scale and the Inventory of Non-Ataxia Signs (INAS). All measures were administered in one session by clinicians as face-to-face interviews in the respective study center.

#### EuroQol 5-Dimensional 3-Level—EQ-5D-3L

The EQ-5D consists of a thermometer-like visual analog scale (EQ-VAS) anchored by 0 (worst health) and 100 (best health) to assess health status at the time of assessment and a 5-item descriptive system with five dimensions: mobility, self-care, usual activities, pain/discomfort, and anxiety/depression. Each item has three levels of responses reflecting no, moderate, and extreme problems [17, 18]. The response pattern in these 5 items can be converted into a single index score, anchored between zero (for death) and 1 (perfect health). For our analyses, EQ-5D-3L indices were calculated using the European value set for use in multinational studies [19].

#### Patient Health Questionnaire 9—PHQ-9

The PHQ-9 is a widely validated self-reported questionnaire designed for use in primary care as a screening instrument for depressive symptoms [20, 21]. The score of the 9-item-questionnaire ranges between 0 and 27 with the following score interpretation: 0 to 4 none to minimal, 5–9 mild, 10–14 moderate, and ≥ 15 severe depression [21].

#### Scale of the Assessment and Rating of Ataxia—SARA

SARA is a valid and reliable clinical rating of the severity of ataxia symptoms. SARA consists of eight items related to gait, stance, sitting, speech, finger-chase test, nose-finger test, fast alternating movements, and heel-shin test [22]. The sum score ranges between 0 (no ataxia) and 40 (most severe ataxia) [22]. We grouped the sum score into the following stages: SARA scores mild 0–9.5, moderate ≤ 19.5, strong ≤ 29.5, and extreme ≤ 40 [23].

#### Inventory of Non-Ataxia Signs—INAS

The INAS asks for a total of 30 items that are categorized into 16 non-ataxia signs. INAS count denotes the number of non-ataxia signs, ranging from zero (absence of non-ataxia signs) to a maximum of 16. A higher INAS count can be interpreted as a more complex extracerebellar disorder [24].

#### Activities of Daily Living (ADL) Part of the Friedreich Ataxia Rating Scale (FARS)

The FARS is a validated rater-based scale [25], comprising a measure of ataxia and ADL subscale and a neurological subscale. The scores of each measure can be added to make a total score ranging from 0 to 36, with higher scores representing more severe impairment. ADL was only assessed in the ESMI study.

### Statistical Analyses

Patients’ socio-demographics (gender, age), age of onset of gait, and clinical characteristics (SARA, INAS, and ADL) were analyzed descriptively. Chi-square tests or *t*-tests have been applied to examine differences between the two genotype groups (SCA 1, 2, and 3 vs. SCA 6).

For the psychometric analysis, the EQ-5D-3L was analyzed in terms of acceptability, distributional properties, convergent validity, known-groups validity, test-retest reliability, and responsiveness to change. No imputation was used to deal with missing data, resulting in complete case analyses.

#### Completeness of Data

The frequency of missing values in both genotype groups for the EQ-5D-3L index and EQ-VAS was used as an indicator for acceptance.

#### Distributional Properties

Mean, standard deviation, minimum and maximum, and floor and ceiling effects were assessed for EQ-5D-3L index and EQ-VAS regarding the two genotype groups. Frequencies of responses per EQ-5D-3L item are reported.

#### Convergent Validity

The association between the EQ-5D-3L dimensions, EQ-5D-3L index, and EQ-VAS with SARA, INAS, ADL, and PHQ-9 were assessed using Spearman’s correlation coefficient. Correlations were interpreted as follows: *r*_sp_ < 0.3 small, 0.3 ≥ *r*_sp_ < 0.5 moderate, and *r*_sp_ ≥ 0.5 high/strong [26, 27]. The sign of the coefficient can be positive as well as negative, indicating the direction of the relationship [28]. We expected higher correlations between EQ-5D dimensions mobility, self-care and usual activity and the SARA score, INAS count and ADL, and the EQ-5D dimensions pain/discomfort and anxiety/depression with the PHQ-9. Lower correlations were expected between mobility, self-care, and usual activity with PHQ-9. For the convergent validity, we recognized all observations from baseline to *T*_3_. Furthermore, we created scatter plots with rugs for inspection of these associations for baseline and *T*_3_. Analyses were conducted separately for each genotype group.

#### Known-Groups Validity

Known-groups validity is the ability to distinguish between different health states and disease groups. We compared the EQ-5D-3L index between two groups of ages (≤ 51 years old, > 51 years old), ataxia severity (SARA score: mild 0–9.5, moderate ≤ 19.5, strong ≤ 29.5, extreme ≤ 40) [23], non-ataxia symptoms (INAS: ≤ 4 symptoms, > 4 symptoms), depression (PHQ-9: minimal ≤ 4, moderate ≤ 9) [21], and the EQ-VAS (low ≤ 60; high > 60). We hypothesized that the EQ-5D-3L index would be lower in older patients, patients with a higher SARA score, a higher INAS count, a higher PHQ-9, and a lower EQ-VAS value in both genotype groups.

#### Test-Retest Reliability

Test-retest reliability is expressed by the ability of a test’s consistency in repeated testing. We tested for consistency of scores over time comparing the EQ-5D-3L’s responses at baseline and *T*_1_ among “stable” patients according to their symptom severity. A patient was considered stable if there were no changes in the SARA score and INAS count within 1 year. For the single-item dimensions of EQ-5D-3L, we calculated Cohens Kappa; for EQ-5D-3L index and EQ-VAS, intraclass correlation coefficients (ICC) were reported. Cohens Kappa values > 0.40 [29] and ICCs values > 0.75 [30] indicate acceptable reliability.

#### Responsiveness

Responsiveness refers to the EQ-5D-3L ability to capture changes in health over time. We analyzed the changes in the data pairs from baseline (*T*_0_) to *T*_1_, *T*_0_ to *T*_2_, and *T*_0_ to *T*_3_. Changes in the EQ-5D-3L index/EQ-VAS were then analyzed separately in subgroups of patients based on the SARA score: group 1 “no changes” in the SARA score (SARA score *T*_0_ = *T*_1_; *T*_0_ = T_2_, and *T*_0_ = *T*_3_); group 2: “deteriorated” in the SARA score (SARA score *T*_0_ < *T*_1_; *T*_0_ < *T*_2_, and *T*_0_ < *T*_3_). Differences in the EQ-5D-3L index and EQ-VAS between time points were divided by baseline standard deviation to produce standardized effect size (SES) or by the pooled standard deviation of change to calculate standardized response means (SRM), calculated with paired sample *t*-tests. We interpreted the effect sizes as < 0.3 small, 0.3 to 0.59 moderate, and ≥ 0.6 large [26] [31]. We expected to observe significant health changes in group 2 and no or minimal changes in group 1. Given the decreasing number of patients over time, we analyzed all patients regardless of genotype groups. SES and SRM are commonly used effect sizes in many validation studies of the EQ-5D [32], supporting the comparison of findings. For the SARA score, SES and SRM were also calculated.

Data were analyzed using IBM SPSS Statistics (Version 21). The R package “ggplot2” was used to create graphs (Version 4.2.1).

## Results

### Sample

Table 1 presents the characteristics of the sample across both genotype groups. Patients were, on average, 49.9 ± 13.7 years old, and 49% were women.

**Table 1:** Characteristics of the patients at baseline with full data of EQ-5D-3L and distributional properties of clinical parameters and patient-reported outcomes

	Total (*n* = 835)	SCA 1–3 (*n* = 728)	SCA 6 (*n* = 107)	*p-value*
Sociodemographic variables				
Sex, % women	49.0	49.5	45.8	0.471*
Age, *M* ± SD; range	49.9 ± 13.7; 14–85	47.7 ± 12.6; 14–84	65.0 ± 10.9; 37–85	< 0.01**
Age of onset, *M* ± SD	39.8 ± 12.5	37.5 ± 11.1	54.3 ± 10.6	< 0.01**
Clinical parameters				
SARA score, M±SD	14.0 ± 8.6 (*n* = 832)	13.8 ± 8.9 (*n* = 725)	15.21 ± 6.8	0.061**
INAS count, *M* ± SD	4.1 ± 2.6 (*n* = 748)	4.4 ± 2.6 (*n* = 656)	1.95 ± 1.6 (*n* = 92)	< 0.01**
ADL, *M* ± SD	9.2 ± 8.3 (*n* = 301)	9.2 ± 8.4 (*n* = 301)	-	
Patient-reported outcomes				
EQ-5D-3L Index				
*M* ± SD	0.65 ± 0.21	0.65 ± 0.22	0.65 ± 0.17	0.726**
Min	0.03	0.03	0.21	-
Max	1.0	1.0	1.0	-
Ceiling effect, % (*n*)	9.9% (*n* = 83)	10.9 (*n* = 79)	3.7 (*n* = 4)	-
Floor effect, % (*n*)	0.6% (*n* = 5)	0.7 (*n* = 5)	-	-
EQ-5D-VAS				
*M* ± SD	63.4 ± 21.2 (*n* = 813)	63.3 ± 21.5 (*n* = 707)	63.8 ± 18.9 (*n* = 106)	0.831**
Min	0	0	20	-
Max	100	100	100	-
Ceiling effect, % (*n*)	3.0 (*n* = 24)	3.3 (*n* = 23)	0.9 (*n* = 1)	-
Floor effect, % (*n*)	0.6 (*n* = 5)	0.7 (*n* = 5)	-	-
PHQ-9				
*M* ± SD	6.7 ± 5.8 (*n* = 786)	6.9 ± 5.8 (*n* = 679)	5.28 ± 5.4 (*n* = 107)	< 0.01**

*M* mean, *SD* standard deviation, *Min* minimum, *Max* maximum, *SARA* Scale for Assessment and Rating of Ataxia, *INAS* Inventory of Non-Ataxia Signs, *ADL* activities of daily living (ADL) part of Friedreich’s Ataxia Rating Scale, *EQ-VAS* EuroQol Visual Analog Scale, *PHQ-9* Patient Health Questionnaire

*Chi-quadrat; **t-test

As known for SCA 6, the age of onset in that group started later in life with an average of 54.3 years compared to SCA 1–3 with 37.5 years. Most patients had a moderate ataxia severity (SARA mean score 14.0) and a mean EQ-5D index of 0.65 in both groups. SCA 6 patients reported lower INAS and a slightly lower PHQ-9.

### Completeness of Data and Distributional Properties

Missing values occurred occasionally for the EQ-5D index (0.8%; *n*=7) and EQ-VAS (3.4%; *n*=29). Supplementary Figure 2 presents responses to the EQ-5D-3L items. The proportion of patients reporting “no problems” in any dimension was 10.9% for SCA 1–3 and 3.7% for SCA 6 (Supplementary Table 2). More than 50% of patients reported no problems with SC and AD at baseline. Only a small proportion of patients reported extreme health-related problems. Table 1 includes the distributional properties of the EQ-5D-3L index and EQ-VAS. The EQ-5D-3L-derived indices ranged from 0.03 to 1.0, with a ceiling effect of 9.9% of overall patients. Ceiling effects of the EQ-VAS occurred in less than 4% of the total sample.

### Convergent Validity

We observed low (*r* < 0.3) to strong (*r* ≥ 0.5) Spearman correlations between EQ-5D-3L dimensions and SARA, ADL, INAS, and PHQ-9. For patients with SCA 1–3, SARA, and ADL correlated strongly and statistically significant (*p* < 0.01) with the physical dimensions (self-care: *r*_SARA_ = 0.596/*r*_ADL_ = 0.689; usual activity: *r*_SARA_ = 0.534/*r*_ADL_ = 0.682) of EQ-5D-3L and low with mental items (pain/discomfort: r_SARA_= 0.120/r_ADL_= 0.280; anxiety/depression: r_SARA_= 0.193/r_ADL_= 0.231; all correlations with *p* < 0.01). Contrary to this, PHQ-9 showed moderate to strong statistically significant (*p* < 0.01) correlations with pain/discomfort (*r*_PHQ-9_ = 0.355) and anxiety/depression (*r*_PHQ-9_ = 0.530). Findings were similar in SCA 6 patients, even though correlations were constantly lower for this group. With respect to INAS, only in SCA 1–3 moderate correlations with *p* < 0.01 were seen to mobility (*r*_INAS_ = 0.410), self-care (*r*_INAS_ = 0.419), and usual activity (*r*_INAS_ = 0.431) while only poor correlations were seen for pain/discomfort (*r*_INAS_ = 0.156) and anxiety/depression (*r*_INAS_ = 0.146) and all dimensions in SCA 6 (*r*_INAS_ < 0.3 with *p* < 0.01 in usual activity, pain/discomfort, and anxiety/depression).

**Fig. 1:**
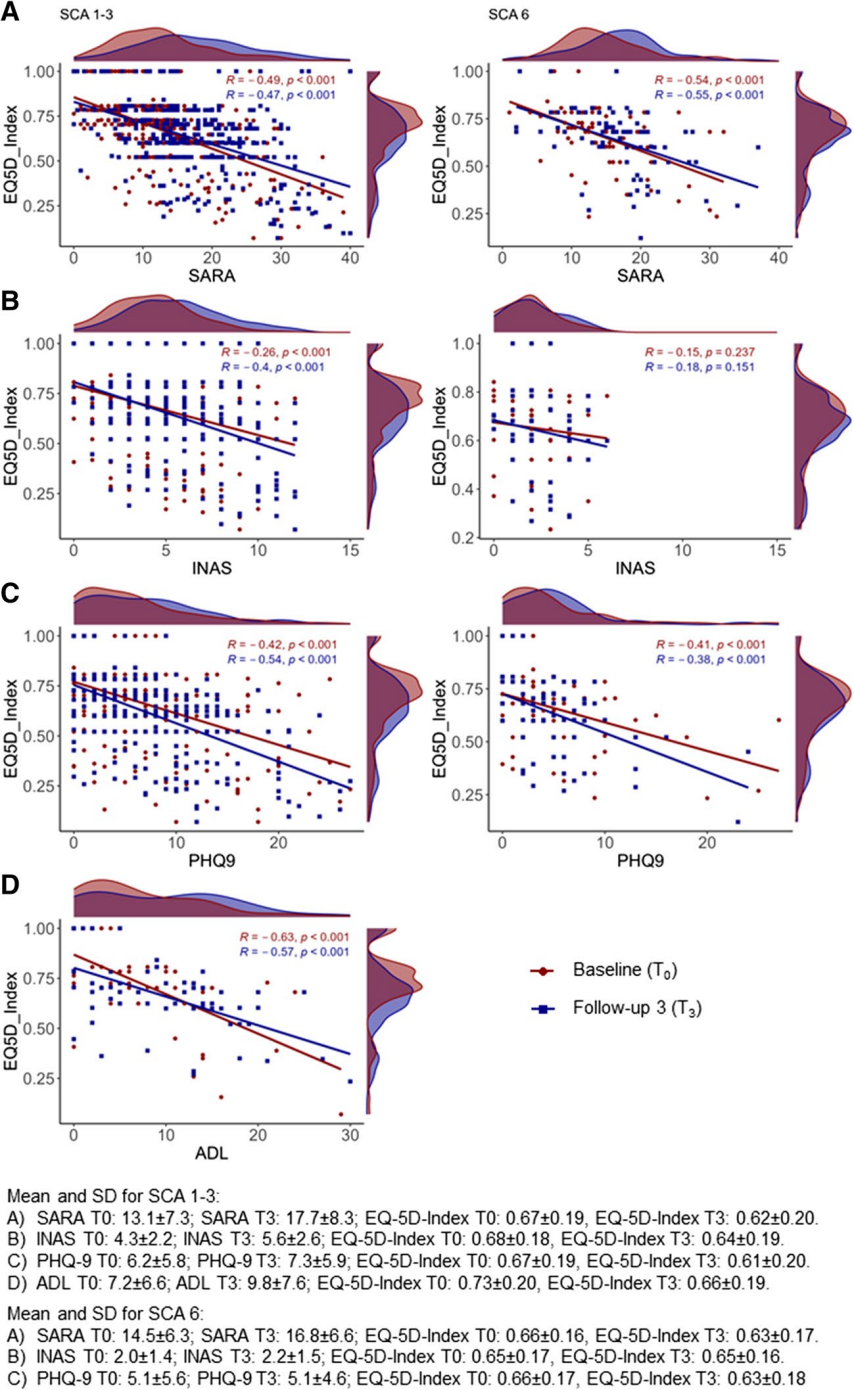
Scatterplots with rugs between the EQ-5D-3L, SARA, INAS PHQ-9, and ADL for both genotype groups

The EQ-5D-3L index showed moderate (*r*_INAS_ = −0.381 and *r*_PHQ-9_ = −0.474) to strong (*r*_SARA_ = −0.562 and *r*_ADL_ = −0.713) correlations (Table 2). EQ-VAS showed a strong correlation with ADL (*r*_ADL_ = −0.507) and a moderate correlation with SARA (*r*_SARA_ = −0.396), INAS (*r*_INAS_ = −0.329) and PHQ-9 (*r*_PHQ-9_ = −0.453) in the entire sample (Table 2). When analyzed by genotype groups, EQ-5D-3L was stronger correlated in patients with SCA 1–3. Correlations in this group were highest for ADL (*r*_ADL_ = −0.713), followed by SARA (*r*_SARA_ = −0.575) and PHQ-9 (*r*_PHQ-9_ = −0.493). EQ-VAS correlated moderately with all instruments. In the group of SCA 6 patients, EQ-5D-3L presented lower correlation coefficients consistently with poor correlations for EQ-5D-3L index and INAS (*r*_INAS_ = −0.230) and EQ-VAS and SARA (*r*_SARA_ = −0.230) while EQ-VAS correlated strongly with PHQ-9 (*r*_PHQ-9_ = −0.517). All correlations were statistically significant. Figure 1 illustrates the correlation between the EQ-5D-3L index and SARA, INAS, PHQ-9, and ADL.

**Table 2:** Convergent validity of the EQ-5D-3L index and EQ-VAS, all observations (*T*_0_, *T*_1_, *T*_2_, *T*_3_)

	Total			SCA 1–3			SCA 6		
	*r*	*n*	*p*-value	*r*	*n*	*p*-value	*r*	*n*	*p*-value
EQ-5D-3L index									
SARA score	−0.562	2528	< 0.01	−0.575	2178	< 0.01	−0.468	350	< 0.01
ADL	−0.713	768	< 0.01	−0.713	768	< 0.01			
INAS count	−0.381	2261	< 0.01	−0.440	1956	< 0.01	−0.230	305	< 0.01
PHQ-9	−0.474	2374	< 0.01	−0.493	2042	< 0.01	−0.376	332	< 0.01
EQ-VAS									
SARA score	−0.396	2460	< 0.01	−0.423	2114	< 0.01	−0.230	346	< 0.01
ADL	−0.507	747	< 0.01	−0.507	747	< 0.01			
INAS count	−0.329	2209	< 0.01	−0.352	1908	< 0.01	−0.379	301	< 0.01
PHQ-9	−0.453	2350	< 0.01	−0.444	2019	< 0.01	−0.517	331	< 0.01

*r* = data are given as Spearman’s correlation coefficient; *n* = number of observations; *T*_0_ = baseline, *T*_1_ = follow-up 1, *T*_2_ = follow-up 2, *T*_3_ = follow-up 3

*SARA* Scale for Assessment and Rating of Ataxia, *INAS* Inventory of Non-Ataxia Signs, *ADL* activities of daily living (ADL) part of Friedreich’s Ataxia Rating Scale, *EQ-VAS* EuroQol Visual Analog Scale, *PHQ-9* Patient Health Questionnaire

### Known-Groups Validity

In SCA 1–3, results fit the assumptions made for differences between known groups according to age, SARA, INAS, PHQ-9, and general health (EQ-VAS). EQ-5D-3L index was lower in older patients, patients with more severe ataxia, with higher INAS, higher likelihood of depression, and lower EQ-VAS (Table 3). In SCA 6, such differences were less pronounced and did not reach significance for age and INAS (Table 3).

**Table 3:** Known-groups validity of the EQ-5D-3L, all observations (*T*_0_ to *T*_3_)

Sub groups	SCA 1–3	*n*	*p*-value	SCA 6	*n*	*p*-value
Age						
≤ 51 years old	0.65 ± 0.21	1272	< 0.01	0.67 ± 0.13	42	0.227
> 51 years old	0.61 ± 0.22	920		0.65 ± 0.18	310	
SARA score						
Mild	0.78 ± 0.16	611	< 0.01	0.77 ± 0.15	57	< 0.01
Moderate	0.65 ± 0.16	951		0.65 ± 0.15	213	
Strong	0.52 ± 0.20	451		0.58 ± 0.16	62	
Extreme	0.35 ± 0.27	165		0.50 ± 0.19	18	
INAS count						
≤ 4 symptoms	0.72 ± 0.18	946	< 0.01	0.65 ± 0.16	344	0.231
> 4 symptoms	0.57 ± 0.21	1010		0.60 ± 0.19	39	
PHQ-9						
Mild	0.69 ± 0.19	1459	< 0.01	0.67 ± 0.16	280	< 0.01
Moderate	0.49 ± 0.20	582		0.52 ± 0.17	52	
EQ-VAS						
High (> 60)	0.72 ± 0.19	1063	< 0.01	0.69 ± 0.15	164	< 0.01
Low (≤ 60)	0.55 ± 0.19	1063		0.59 ± 0.16	183	

SARA score: mild 0–9.5, moderate ≤ 19.5, strong ≤ 29.5, extreme ≤ 40, PHQ-9: mild 0–9; moderate 10–14

*n* number of observations, *SARA* Scale for Assessment and Rating of Ataxia, *INAS* Inventory of Non-Ataxia Signs, *ADL* activities of daily living (ADL) part of Friedreich’s Ataxia Rating Scale, *EQ-VAS* EuroQol Visual Analog Scale, *PHQ-9* Patient Health Questionnaire

### Test-Retest Reliability

Among 27 patients classified as stable at 12 months after baseline according to SARA, Kappa statistics of all EQ-5D-3L items was > 0.4, demonstrating acceptable test-retest reliability. ICC calculation of the EQ-5D-3L (0.95) index and the EQ-VAS (0.88) indicated sufficient reliability.

### Responsiveness

Clinically stable patients (no change in SARA score) showed no responsiveness for the EQ-5D-3L index and EQ-VAS. However, in the patient group with an increase in the SARA score over time (SARA score deteriorated), both the EQ-5D-3L index and the EQ-VAS differed between baseline and follow-ups with almost none to small effect sizes. SES varied from 0.08 (*T*_0_ to *T*_1_) to 0.29 (*T*_0_ to *T*_3_) for the EQ-5D-3L index and 0.17 (*T*_0_ to *T*_1_) to 0.34 (*T*_0_ to *T*_2_) for the EQ-VAS. SRM ranged from 0.09 (*T*_0_ to *T*_1_) to 0.27 (*T*_0_ to *T*_2_/*T*_3_) for the EQ-5D-3L index and 0.15 (*T*_0_ to *T*_1_) to 0.33 (*T*_0_ to *T*_2_) for the EQ-VAS, respectively (Table 4). Regarding the solely observation of the SARA score, we found small (*T*_0_ to *T*_1_: 0.2_SES_ and 0.2_SRM_) to moderate (*T*_0_ to *T*_2_: 0.40_SES_ and 0.39_SRM_; *T*_0_ to *T*_3_: 0.59_SES_ and 0.56_SRM_) changes in health for both effect sizes.

**Table 4:** Responsiveness of EQ-5D-3L and EQ-VAS baseline (*T*_0_) to *T*_1_, *T*_2_, and *T*_3_; divided into SARA score “no change” and SARA score “deteriorated”

Instrument	SARA score no change					SARA score deteriorated				
	*n*	Mean Δ	*p*-value	SES	SRM	*n*	Mean Δ	*p*-value	SES	SRM
*T*_0_ to *T*_1_										
Index	77	0.02	0.185	0.08	0.15	463	0.02	0.06	0.08	0.09
EQ-VAS	75	1.8	0.477	−0.09	−0.08	435	3.29	< 0.01	0.17	0.15
*T*_0_ to *T*_2_										
Index	38	−0.01	0.606	0.08	0.08	450	−0.05	< 0.01	0.25	0.27
EQ-VAS	36	3.3	0.286	0.17	0.18	434	−7.1	< 0.01	0.34	0.33
*T*_0_ to *T*_3_										
Index	17	0.0002	0.995	0.001	0.002	367	−0.05	< 0.01	0.29	0.27
EQ-VAS	17	−4.8	0.310	0.25	0.25	353	−6.48	< 0.01	0.27	0.25

*SES* standardized effect size, *SRM*, standardized response mean, *3L* EQ-5D-3L, *VAS* EQ-VAS, *SARA* Scale for Assessment and Rating of Ataxia

## Discussion

To the best of our knowledge, this is the first longitudinal study reporting specific evidence on the psychometric properties of the EQ-5D-3L in patients with SCA. Results of this study fill the gap of inconclusive evidence by showing that the EQ-5D-3L demonstrates acceptability, reliability, good discriminative ability, moderate to strong convergent validity, and small (EQ-5D-3L indices) to moderate (EQ-VAS with regard to ataxia severity) responsiveness to health changes in measuring HRQoL among patients with SCA 1, 2, 3, and 6. Our analysis supports the NICE recommendations [12] to gain information about the EQ-5D-3L performance in rare diseases. It provides evidence that the EQ-5D-3L could be an appropriate measure to capture the patients’ HRQoL of SCA patients for use in clinical trials, in health economic evaluations, and in further research understanding HRQoL in people with that rare disease.

### Distribution Properties

The extent of non-item response with less than 5% (EQ-5D index = 0.8% and EQ-VAS = 3.4%) is very low and acceptable. Our results are consistent with the findings of other studies regarding item-based ceiling effects. Bolzan et al. [5] analyzed HRQoL in the pre-ataxic phases in a Brazilian sample of SCA 3 patients using the EQ-5D-3L. The results of this study demonstrated the highest proportion of “no problems” in self-care (73.9%) and the lowest in mobility (13%). Responses of our patients are in line with Bolzan et al. [5], occurring with the highest ceiling effects in self-care (only SCA 1–3) and the lowest in mobility (for both groups). Maas et al. [10] analyzed the discordance between the EQ-5D-5L and physician-rated motor symptom severity in early-to-middle-stage SCA 3, revealing a comparable high “no problem” area in SC (70%). Ceiling effects of the EQ-5D-3L index are well known and highly differed in percentage in terms of the analyzed setting [32–34]. We detected acceptable ceiling effects of the EQ-5D-3L with 10.9% in SCA 1–3 and 3.7% in SCA 6 patients with an index range of 0.03 to 1.0 (SCA 1–3) and 0.21 to 1.0 (SCA 6). Bolzan et al. [5] showed that SCA patients differed in their HRQoL from healthy individuals, especially in the EQ-5D mobility dimension. Item responses of our sample show nearly identical results of a reduced HRQoL dominantly in the dimension mobility with a proportion of > 70% reporting at least some problem in both SCA groups. However, due to the evidence that the 3L tends to overestimate health problems resulting in a biased index score [35] and missing HRQoL data of SCA patients, we cannot conclusively state that our findings present the full range of health states in SCA patients.

### Validity

It is well known that the EQ-5D-3L can be a useful tool to screen symptoms of anxiety and depression compared to the PHQ-9 [36], also evaluated as appropriate for use in SCA [37]. Symptoms of depression are common in SCA patients

[38]. According to our PHQ-9 screening results, no patient in our survey met the criterion for clinically relevant depression. Nevertheless, we found moderate to strong correlations between EQ-5D-3L/EQ-VAS and the PHQ-9, which underlines the possibility of using EQ-5D as a substitute for capturing the patients’ mood.

Several validation studies in different settings and patient populations found at least moderate associations between the EQ-5D and disease-specific instruments and clinical assessments [39–41]. Our study also confirmed moderate to strong correlations between EQ-5D-3L-dimensions, EQ-5D-3L index, and EQ-VAS with the disease-specific ratings of SARA, INAS, and ADL. This supports our hypothesis of convergent validity testing in an adequate correlation structure. Including all observations (*T*_0_ to *T*_3_) in the validity analysis may lead to within-subject effects as individuals with more data points are included. A separate analysis of all observations showed almost equally high correlation coefficients, indicating robust results when all time points (*T*_0_ to *T*_3_) are considered (Supplementary Table 3). The EQ-5D-3L can also successfully differentiate among different stages of age and health components (SARA, INAS, PHQ-9, EQ-VAS). In addition to clinically relevant parameters in SCA patients, the EQ-5D-3L represented strong convergent validity and was useful in assessing a comprehensive picture of the patient’s health status.

### Test-Retest Reliability

A substantial body of literature analyzed the EQ-5D-3L’s reliability. We studied the test-retest reliability of the EQ-5D-3L by calculating Cohen’s Kappa and ICCs for stable patients from baseline to *T*_1_. Both coefficients passed the threshold for tolerable reliability (Kappa range: 0.44–0.93; ICC: > 0.8). Our results replicate international findings, including those for rare diseases [39, 42], suggesting that the EQ-5D-3L shows acceptable reproducibility in SCA (Supplementary Table 4). Nevertheless, the results must be interpreted with caution and be seen more as a tendency regarding the small sample size of stable patients (*N* < 30).

### Responsiveness

The EQ-5D-3L’s ability to detect changes in health varies greatly by setting and patient group. Shah et al. [39] reported poor responsiveness of EQ-5D-3L in patients with rare lung diseases; whereas, Golicki et al. [43] documented a moderate to good ability to detect changes in health in stroke patients. Compared to other HRQoL measures, the EQ-5D, especially the three-level version, is less responsive [32]. As EQ-5D indices and EQ-VAS values in patients deteriorated similarly to ataxia severity (SARA score), we can assume responsiveness to changes in ataxia severity in SCA 1–3 and SCA 6. In that case, we have to consider that SCA is a slowly progressive neurodegenerative disease, which means that changes in functional and mental health over the observed time are expectedly small. Therefore, we did not expect to see strong effect sizes for EQ-5D-3L.

### Strengths and Limitations

The strength of our study is primarily in the study design and the included large population group. While analyses of rare diseases are often associated with a small number of cases in a cross-sectional design, we analyzed a longitudinal data set with a large sample of *n* = 842 SCA patients with multiple observations. Nevertheless, we know that the validation of the EQ-5D-3L in SCA patients is confronted with limitations. First of all, the data set did not include additional well-established HRQoL measurements. We therefore could not compare the EQ-5D-3L to other HRQoL instruments with comparable constructs, which limits the generalizability of the results. Another limitation is the design of the measurement time points, defined as a 1-year follow-up. The 1-year distance between the time points can be interpreted as long, not capturing variations in the patient’s HRQoL. The slow progression of SCA diseases and the low clinical and patient-reported changes in health status support the choice of 1-year follow-ups. Our test-retest reliability analysis must be interpreted with caution; it relies on only a small subgroup of subjects classified as clinically stable. To obtain robust results, it would be favorable to administer the EQ-5D-3L, for example, on two consecutive days. However, our data deliver a good estimate of score stability when applied in larger intervals, which is the common clinical setting. There is a probability that combining SCA 1, 2, and 3 into one group will influence the interpretation of the results. Despite this, studies revealed that the clinical picture of these three SCA types is very similar [16, 17], supporting the grouping of these three SCA disorders. Finally, the EQ-5D-3L is increasingly replaced by the 5L version. Several studies reported an improved sensitivity with the 5L version, reducing ceiling and floor effects and improving other psychometric properties [32, 34]. We are conscious that using the 3L version in our survey leads to the risk of a reduced interpretation of the measurement properties. Due to this, further research should evaluate if an extension from three to five levels would influence the performance of the EQ-5D in SCA patients [44].

## Conclusion

The psychometric analysis demonstrates that the EQ-5D-3L is an acceptable, valid, and reliable instrument for patients with SCA 1, 2, 3, and 6 and shows overall low responsiveness. Thus, the EQ-5D-3L could be used regularly in economic evaluations in this rare disease area and as an additional instrument to the disease-specific HRQoL measures in SCA clinical research [45]. Nevertheless, further research is needed to evaluate if the 5L version would improve the psychometric properties of the EQ-5D in SCA.

### Supplementary Information

ESM 1 (DOCX 210 kb)

## Data Availability

Data is available on request.
