# Supplementary material for: Health-Related Quality of Life in Patients with Spinocerebellar Ataxia: a Validation Study of the EQ-5D-3L
Source: Cerebellum. 2023 Sep 15;23(3):1020–30. doi: 10.1007/s12311-023-01597-3 (PMC11102408; doi:10.1007/s12311-023-01597-3)
Supplement: Supplementary file 1 — (DOCX 210 kb) [file 12311_2023_1597_MOESM1_ESM.docx]

Supplementary Tables

|  | **T_0_ -T_1_** | **T_1_ –T_2_** | **T_2_ –T_3_** |
| --- | --- | --- | --- |
| M±SD | 400.3±91.6 | 394.4±120.7 | 398.5±119.3 |
| Median | 382 | 373 | 371 |
| IQR | 360-417 | 353-402 | 350-413 |

Supplementary Table 1: Time between the observations in days

M: Mean; SD: Standard Deviation; IQR: Interquartile range

Supplementary Table 2: Percentage of patients with ceiling effects for each EQ-5D-3L dimension

| **EQ-5D dimension** | **SCA 1-3 % (n=728)** | **SCA 6 % (n=107)** |
| --- | --- | --- |
| Mobility | 19.6 | 12.1 |
| Self-care | 64.8 | 60.7 |
| Usual activities | 39.1 | 21.5 |
| Pain/Discomfort | 49.7 | 62.6 |
| Anxiety/Depression | 58.1 | 57.9 |
| **Over all** '**11111**' **%** | 10.9 | 3.7 |

'11111': no problems in all dimensions. Each of the five dimensions consists of three response options, numbered with (1) no problems, (2) some problems, (3) extreme problems, indicating of no problems with ‘11111’.

Supplementary Table 3: Convergent validity of the EQ-5D-3L index and EQ-VAS; baseline to follow-up 3

|  | **Total** | **n** | **p-value** | | **SCA 1-3** | | **n** | **p-value** | **SCA 6** | **n** | **p-value** |
| --- | --- | --- | --- | --- | --- | --- | --- | --- | --- | --- | --- |
| **Baseline** | | | | | | | | | | | |
| **EQ-5D-3L index** | |  |  | |  | |  |  |  |  |  |
| SARA score | -0.603 | 832 | <0.01 | | -0.612 | | 725 | <0.01 | -0.500 | 107 | <0.01 |
| ADL | -0.770 | 301 | <0.01 | | -0.770 | | 301 | <0.01 |  |  |  |
| INAS count | -0.404 | 748 | <0.01 | | -0.473 | | 656 | <0.01 | -0.255 | 92 | 0.014 |
| PHQ-9 | -0.411 | 786 | <0.01 | | -0.434 | | 679 | <0.01 | -0.301 | 107 | <0.01 |
| **EQ-VAS** |  |  | |  | |  |  |  |  |  |  |
| SARA score | -0.414 | 810 | <0.01 | | -0.434 | | 704 | <0.01 | -0.281 | 106 | <0.01 |
| ADL | -0.504 | 293 | <0.01 | | -0.504 | | 293 |  |  |  |  |
| INAS count | -0.293 | 730 | <0.01 | | -0.324 | | 639 | <0.01 | -0.319 | 91 | <0.01 |
| PHQ-9 | -0.389 | 774 | <0.01 | | -0.393 | | 668 | <0.01 | -0.381 | 106 | <0.01 |
| **Follow-up 1** | | | | | | | | | | | |
| **EQ-5D-3L index** |  |  |  | |  | |  |  |  |  |  |
| SARA score | -0.541 | 695 | <0.01 | | -0.559 | | 612 | <0.01 | -0.357 | 83 | <0.01 |
| ADL | -0.714 | 249 | <0.01 | | -0.714 | | 249 | <0.01 |  |  |  |
| INAS count | -0.363 | 585 | <0.01 | | -0.414 | | 523 | <0.01 | -0.197 | 62 | 0.125 |
| PHQ-9 | -0.464 | 645 | <0.01 | | -0.471 | | 570 | <0.01 | -0.464 | 75 | <0.01 |
| **EQ-VAS** |  |  |  | |  |  |  |  |  |  |  |
| SARA score | -0.365 | 672 | <0.01 | | -0.397 | | 591 | <0.01 | -0.134 | 81 | 0.231 |
| ADL | -0.484 | 240 | <0.01 | | -0.484 | | 240 |  |  |  |  |
| INAS count | -0.300 | 566 | <0.01 | | -0.300 | | 506 | <0.01 | -0.482 | 60 | <0.01 |
| PHQ-9 | -0.466 | 637 | <0.01 | | -0.454 | | 562 | <0.01 | -0.552 | 75 | <0.01 |
| **Follow-up 2** | | | | | | | | | | | |
| **EQ-5D-3L index** |  |  |  | |  | |  |  |  |  |  |
| SARA score | -0.547 | 567 | <0.01 | | -0.561 | | 484 | <0.01 | -0.477 | 83 | <0.01 |
| ADL | -0.64 | 155 | <0.01 | | -0.64 | | 155 | <0.01 |  |  |  |
| INAS count | -0.376 | 525 | <0.01 | | -0.417 | | 449 | <0.01 | -0.210 | 76 | 0.068 |
| PHQ-9 | -0.528 | 532 | <0.01 | | -0.549 | | 456 | <0.01 | -0.389 | 76 | <0.01 |
| **EQ-VAS** |  |  |  | |  |  |  |  |  |  |  |
| SARA score | -0.390 | 556 | <0.01 | | -0.435 | | 474 | <0.01 | -0.114 | 82 | 0.309 |
| ADL | -0.523 | 151 | <0.01 | | -0.523 | | 151 |  |  |  |  |
| INAS count | -0.385 | 516 | <0.01 | | -0.401 | | 441 | <0.01 | -0.413 | 75 | <0.01 |
| PHQ-9 | -0.510 | 529 | <0.01 | | -0.486 | | 453 | <0.01 | -0.626 | 76 | <0.01 |
| **Follow-up 3** | | | | | | | | | | | |
| **EQ-5D-3L index** |  |  |  | |  | |  |  |  |  |  |
| SARA score | -0.475 | 434 | <0.01 | | -0.469 | | 357 | <0.01 | -0.550 | 77 | <0.01 |
| ADL | -0.593 | 63 | <0.01 | | -0.593 | | 63 | <0.01 |  |  |  |
| INAS count | -0.342 | 403 | <0.01 | | -0.405 | | 328 | <0.01 | -0.260 | 75 | 0.024 |
| PHQ-9 | -0.526 | 411 | <0.01 | | -0.547 | | 337 | <0.01 | -0.382 | 74 | <0.01 |
| **EQ-VAS** |  |  |  | |  |  |  |  |  |  |  |
| SARA score | -0.375 | 422 | <0.01 | | -0.373 | | 345 | <0.01 | -0.381 | 77 | <0.01 |
| ADL | -0.525 | 63 | <0.01 | | -0.525 | | 63 | <0.01 |  |  |  |
| INAS count | -0.327 | 397 | <0.01 | | -0.377 | | 322 | <0.01 | -0.350 | 75 | <0.01 |
| PHQ-9 | -0.461 | 410 | <0.01 | | -0.453 | | 336 | <0.01 | -0.522 | 74 | <0.01 |

Data are given as Spearman's correlation coefficient; n= number of observations; SARA: Scale for Assessment and Rating of Ataxia; INAS: Inventory of Non-Ataxia Signs; ADL: Activities of Daily Living (ADL) part of Friedreich's ataxia rating scale; EQ-VAS: EuroQol Visual Analog Scale; PHQ-9: Patient Health Questionnaire

Supplementary Table 4: Test-Retest Reliability

|  | **n** | **Kappa** | **p-value** |
| --- | --- | --- | --- |
| Mobility | 27 | 0.93 | <0.01 |
| Self-Care | 27 | 0.68 | <0.01 |
| Usual Activities | 27 | 0.52 | <0.01 |
| Pain/ Discomfort | 27 | 0.57 | <0.01 |
| Anxiety/Depression | 27 | 0.44 | <0.01 |
|  |  | **ICC** |  |
| EQ-5D-3L | 27 | 0.95 | <0.01 |
| EQ-VAS | 25 | 0.88 | <0.01 |

ICC: Intraclass correlation coefficient; n= number of observations

Supplementary figures

Supplementary Figure 1: Flow-chart of patients at baseline and follow-ups (FU)


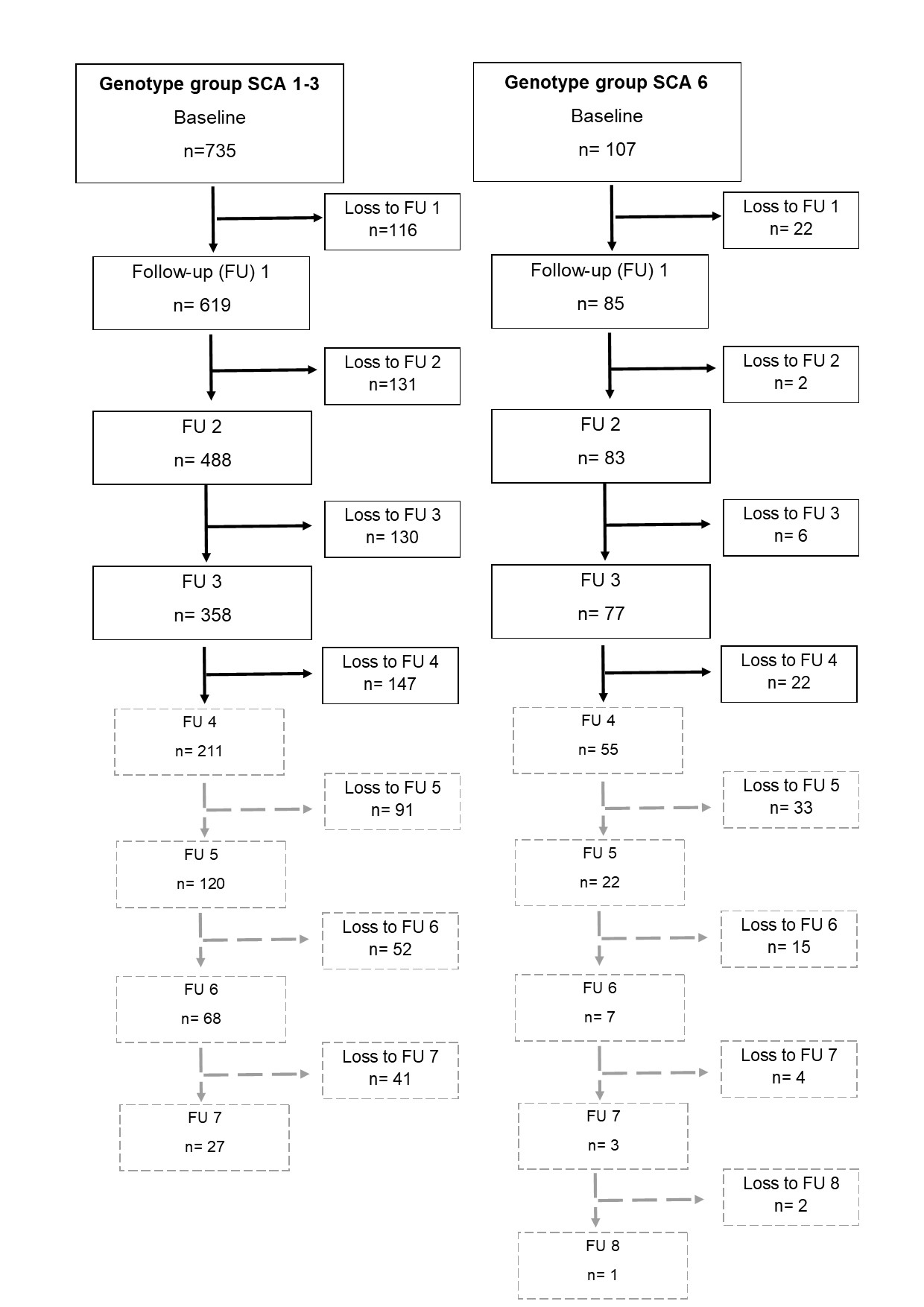


Black bordered fields mark all included follow-ups in our analysis. Fields outlined in grey mark all excluded follow-ups.

Supplementary Figure 2: Distribution of responses to the EQ-5D-3L items MO, SC, UA, PD and AD (in %)

MO: Mobility, SC: Self-care, UA: Usual activities, PD: Pain/Discomfort, AD: Anxiety/Depression
